# Simulation in the Development of Non-Technical Skills in Cardiopulmonary Resuscitation: A Scoping Review

**DOI:** 10.3390/nursrep16100364

**Published:** 2026-10-13

**Authors:** Leila Sales, Bárbara Marques, Ângela Mendes, Florbela Machado, Inês Lopes, Rita Marques

**Affiliations:** 1 CrossI&D Lisboa Research Center, Escola Superior de Saúde da Cruz Vermelha Portuguesa, 1300-125 Lisbon, Portugal; lsales@esscvp.eu (L.S.); rmarques@esscvp.eu (R.M.); 2 Unidade de Investigação em Ciências da Saúde: Enfermagem, Escola de Enfermagem de Coimbra, 3045-043 Coimbra, Portugal; 3 Departamento de Enfermagem, Escola Superior de Saúde da Cruz Vermelha Portuguesa, 1300-125 Lisbon, Portugal; angelamendes9740@esscvp.eu (Â.M.); florbelamachado9922@esscvp.eu (F.M.); ineslopes9760@esscvp.eu (I.L.); 4 Hospital Curry Cabral, Unidade Local de Saúde São José, 1069-166 Lisbon, Portugal; 5 Instituto Português de Oncologia de Lisboa Francisco Gentil, 1099-023 Lisbon, Portugal; 6 Hospital Egas Moniz, Unidade Local de Saúde Lisboa Ocidental, 1349-019 Lisbon, Portugal; 7 Santarém Hospital, Unidade Local de Saúde da Lezíria, 2005-177 Santarém, Portugal; 8 Centro de Investigação Interdisciplinar em Saúde, Universidade Católica Portuguesa, 1649-023 Lisbon, Portugal

**Keywords:** simulation training, patient care team, cardiopulmonary resuscitation, non-technical skills, communication, nursing

## Abstract

**Background:** Cardiopulmonary resuscitation (CPR) requires a coordinated and effective response, with non-technical skills (NTS) playing an important role in patient safety. Deficiencies in these skills may contribute to preventable clinical errors. **Objectives:** To map the scientific evidence on the contribution of simulation to the development of NTS among healthcare teams in the context of CPR. **Methods:** This scoping review followed JBI guidance and PRISMA-ScR reporting recommendations. MEDLINE, CINAHL, Nursing & Allied Health Collection: Comprehensive and the Cochrane Central Register of Controlled Trials were searched via EBSCOhost up to 7 July 2026. Eligibility followed Population, Concept and Context criteria; two reviewers independently selected and charted the evidence. **Results:** The 27 included sources yielded three themes: simulation as a strategy for skill development; NTS in CPR; and complementary educational interventions. Communication was the most frequently reported NTS, alongside leadership, teamwork, situational awareness, and decision-making. Reported improvements varied across sources and skills, and some diminished or were only partially retained over time. Debriefing was addressed in 21 sources (77.8%). Evidence on complementary technologies remained preliminary, with some tools lacking formal evaluation or validation. **Conclusions:** The findings provide a descriptive map of heterogeneous evidence and suggest that simulation may support NTS development, particularly with structured debriefing. They do not establish effectiveness or effects on clinical outcomes. Robust quantitative and longitudinal studies using validated measures are needed to confirm observed trends and assess retention, transfer to practice, team performance, patient safety, and clinical outcomes.

## 1. Introduction

Cardiopulmonary resuscitation (CPR) refers to the sequence of interventions that are essential for restoring blood circulation and oxygenation of vital organs in situations of cardiopulmonary arrest (CPA) [1]. CPA is a sudden event characterised by the abrupt interruption or failure of cardiac and respiratory functions. The causes of CPA are varied and are most frequently associated with severe cardiac rhythm disturbances and acute myocardial infarction. However, this event may also occur in situations of choking, airway obstruction by a foreign body, or drowning [2].

The annual incidence of in-hospital cardiac arrest (IHCA) in Europe ranges from 1.5 to 2.8 per 1000 hospital admissions; however, in the United States of America (USA), the available data indicate a higher incidence, ranging from six to seven IHCA events per 1000 admissions. The survival rate to hospital discharge is approximately 25% in the USA and ranges from 15% to 34% in Europe. In both settings, survival is influenced by factors such as the location of the arrest, the initial cardiac rhythm, and the level of monitoring at the time of collapse. Among survivors, impairments that may compromise quality of life and hinder a return to usual activities are frequently identified [3].

Regardless of the cause, this is a clinical emergency that requires a rapid, effective and coordinated response from the healthcare team. It is essential to ensure that the care provided is tailored to the person’s needs, with the aim of restoring vital functions, preventing complications, and minimising disability [2,4,5]. Therefore, to support high-quality care, robust and continuous preparation is crucial, and simulation training may contribute to the safe performance of healthcare teams [4].

Simulation has been widely recognised as a strategy, as it enables exposure to complex clinical scenarios and promotes reflection through structured debriefing, facilitating critical and adaptive development [6,7]. It allows clinical skills to be acquired and/or developed without compromising the safety of the person receiving care. However, the skills acquired and/or developed through this training tool encompass not only technical aspects, but also elements related to clinical reasoning, decision-making, and effective communication [8]. Simulation is intended to facilitate safer preparation for managing highly complex situations, such as CPA, in which team coordination and stress management are determining factors for the success of the intervention [4].

The literature describes several classifications of simulation as an educational tool, which may be categorised according to the level of fidelity and the setting in which it is implemented. In this context, a distinction is made between low-fidelity simulation, which uses simple manikins or devices for isolated technical skills (TS) training [9]; medium-fidelity simulation, which incorporates some automated physiological responses and simplified clinical scenarios [10]; and high-fidelity simulation, which uses advanced computerised manikins or standardised actors capable of responding in real time to the team’s interventions, thereby promoting a high level of technical and emotional realism [11]. In addition, other modalities include virtual and augmented reality, which use immersive digital environments for the training of clinical and non-technical skills (NTS) [12]; in situ simulation, conducted in the real clinical setting with the local team, enabling contextualised training and the identification of organisational weaknesses [13]; and laboratory-based simulation, carried out in specialised and controlled centres, promoting scenario standardisation and technical rigour in learning [4,14]. However, it should be noted that each type of simulation offers distinct advantages and that the choice of modality and desired level of fidelity depends not only on the learning objectives but also on the resources available.

Simulation, as an educational strategy, should be grounded in robust theoretical models that support its structure and educational intent. Among the most widely used models is Jeffries’ Simulation Theory, which, following successive updates, came to incorporate seven fundamental components: context, theoretical background, simulation design, simulation experience, facilitator, participant, and outcomes [10,15,16]. These interdependent elements guide the planning, implementation, and evaluation of clinical simulation, promoting structured and meaningful learning experiences centred on skills development.

Based on this theoretical model, clinical simulation can be understood as providing a safe and realistic environment in which professionals can develop and refine skills that are essential to clinical practice, within a controlled setting. In addition to TS, this educational strategy also enables the training of NTS, understood as a set of cognitive, social and interpersonal skills that are fundamental to effective team performance and the safety of the care provided. These include rapid and effective decision-making, collaborative teamwork, clear and objective communication, team leadership and coordination, stress management in high-pressure situations, situational awareness (SA), and the ability to take initiative in optimising care for critically ill patients [5,17].

Team responses during CPR may also be considered, cautiously, through the bystander effect described by Darley and Latané, according to which the presence of others may diffuse individual responsibility for intervening in an emergency [18]. This framework was originally developed in relation to untrained bystanders and should not be assumed to operate identically among healthcare professionals with defined clinical responsibilities. Nevertheless, it provides a complementary psychosocial perspective for considering how unclear leadership and role allocation may hinder coordinated action when several professionals are present. In hospital resuscitation teams, identifiable leadership and clear communication of roles have been described as relevant to team functioning [19]. Within simulation-based education, this perspective may therefore help explain the relevance of practising leadership, role allocation, communication and decision-making, complementing Jeffries’ Simulation Theory as an interpretive framework for selected team dynamics during CPR.

In this context, the relevance of the present study should be highlighted, considering the fact that CPR takes place in highly complex and high-pressure environments in which multiple stress-inducing factors are present, requiring highly effective teamwork. Recognising that the quality and safety of care remain priorities within healthcare services and considering that the literature identifies failures or deficiencies in NTS as one of the main causes of preventable errors in clinical practice, the importance of understanding, developing, and training these skills in order to reduce harm and improve outcomes in situations of CPA becomes evident [8].

Although several studies have explored the use of simulation to develop NTS in CPR, the available evidence remains heterogeneous and fragmented regarding the NTS addressed, the educational strategies employed, and the characteristics of the simulation interventions. In this context, a scoping review was considered the most appropriate methodological approach to systematically map the existing evidence, identify the main concepts, characterise the available literature, and highlight knowledge gaps to inform future research and educational practice [20]. Given the increasing use of simulation as an educational strategy in emergency and critical care settings, this study aimed to map the scientific evidence regarding the contribution of simulation to the development of NTS among healthcare teams in the context of cardiopulmonary resuscitation.

## 2. Methods

### 2.1. Review Design and Methodological Rationale

Before undertaking this review, a preliminary search of the Medical Literature Analysis and Retrieval System Online (MEDLINE) was conducted to identify existing evidence syntheses addressing the same combination of population, concept, and context. No review was identified that specifically mapped the use of clinical simulation for the development of NTS among qualified healthcare teams involved in CPR. More recent evidence syntheses have examined simulation-based NTS training in undergraduate medical education or across interprofessional healthcare teams and multiple clinical fields [21,22]. However, these reviews addressed either a different population or a broader clinical context and did not specifically map the range of simulation modalities, NTS domains, educational components, assessment approaches, and reported outcomes relevant to healthcare teams involved in CPR. This constituted the specific knowledge gap addressed by the present review. Evidence concerning undergraduate teaching relates to a population outside the predefined scope of this review, as undergraduate students were explicitly excluded.

A scoping review was selected because the review question sought to map the breadth and characteristics of the available evidence across different simulation modalities, professional groups, NTS domains, educational interventions, study designs, and outcome measures [20,23].

In accordance with the JBI methodology, the process included defining the objectives and research question, identifying the inclusion criteria, describing the search strategy, selecting the evidence, extracting the data, analysing the evidence, and presenting and synthesising the results.

A review protocol was developed before the review commenced to guide its conduct and was subsequently made publicly available on the Open Science Framework to enhance transparency and provide public access to the methods established a priori. It can be accessed at https://doi.org/10.17605/OSF.IO/BYRP5 (accessed on 4 October 2026).

### 2.2. Eligibility Criteria

The formulation of the research question and the definition of the eligibility criteria were guided by the PCC framework: Population (P), Concept (C), and Context (C). Accordingly, the following research question was defined: “What is the contribution of simulation to the development of non-technical skills among healthcare teams in the context of cardiopulmonary resuscitation?”

The inclusion criteria established for this review were as follows: Population (P)—healthcare teams, namely physicians, nurses, and other professionals qualified to perform CPR and directly involved in the management of patients experiencing CPA; Concept (C)—NTS, including communication, decision-making, leadership, time management, SA, and teamwork; and Context (C)—clinical simulation involving CPR scenarios.

The following exclusion criteria were established: Population (P)—evidence involving undergraduate students and healthcare assistants; Concept (C)—evidence involving out-of-hospital cardiac arrest; and Context (C)—simulations that did not specifically involve CPA scenarios.

Eligible sources were required to report original data relevant to the predefined Population, Concept, and Context, regardless of methodological design. Secondary evidence syntheses, including narrative reviews, were not included as sources of evidence, although they could be used to support the theoretical background or contextualise the discussion. During the database searches, the “Full Text” limiter was applied across all databases; therefore, only records identified by the platforms as having full text available were retrieved for screening. Evidence published in Portuguese, Spanish, and English were included, considering Portuguese was the authors’ native language; Spanish was included as it is one of the most widely spoken languages worldwide, and English was included as it is the predominant language of international scientific research [24,25,26]. This language selection was intended to broaden the coverage of research accessible to the review team. No date restrictions were applied, allowing evidence to be mapped across the full period covered by the selected databases [20].

### 2.3. Search Strategy

The literature search was conducted through the EBSCOhost platform in four electronic databases: MEDLINE, Cumulative Index to Nursing and Allied Health Literature (CINAHL), Nursing & Allied Health Collection: Comprehensive, and the Cochrane Central Register of Controlled Trials. Nursing & Allied Health Collection: Comprehensive was selected and searched as a distinct database within EBSCOhost and was not integrated within CINAHL.

In accordance with the JBI methodological guidance, database selection was guided by the review question, the eligibility criteria, and the disciplinary areas encompassed by the Population, Concept, and Context framework, rather than by a predetermined numerical threshold [20]. MEDLINE provides broad coverage of biomedical, clinical, and health sciences research [27], whereas CINAHL offers specialised coverage of nursing and allied health research [28]. Nursing & Allied Health Collection: Comprehensive was included to provide additional access to research relating to nursing and allied health disciplines. The Cochrane Central Register of Controlled Trials was selected because of its specialised coverage of reports of randomised and quasi-randomised controlled trials evaluating healthcare interventions, including potentially relevant simulation-based and educational interventions [29,30].

PubMed was not included as a separate resource in the prespecified database set. MEDLINE, accessed through EBSCOhost, served as the principal database for biomedical and clinical research within a database set designed to provide complementary coverage of the disciplinary areas addressed by the review.

Grey literature sources and trial registries were not systematically searched as part of the formal evidence identification process. Any additional grey literature sources consulted to support the theoretical background presented in the Introduction and to contextualise the interpretation of the findings in the Discussion were neither assessed against the eligibility criteria nor subjected to the study selection or data-charting processes and were not included as sources of evidence in the review.

No date restrictions were applied, and searches covered the period from database inception to July 2026, with the final search conducted on 7 July 2026.

The EBSCOhost “Full Text” limiter was applied to each database search, as reported in Supplementary File S2.

To ensure a rigorous and comprehensive search, Medical Subject Headings (MeSH) were initially identified and complemented with natural-language terms. This option was justified by the specificity of the topic and the need to include terms frequently used in titles and abstracts but not always formally indexed. Accordingly, the terms “Soft Skill*” and “Non-Technical Skill*” were included to increase the sensitivity of the search. Broader terms, including “Clinical Competenc*” and “Clinical Skill*”, were also intentionally included to maximize search sensitivity and capture studies in which NTS were described using broader competency-related terminology. Records addressing exclusively technical skill acquisition without a clear focus on NTS were subsequently excluded during the screening process according to the predefined eligibility criteria. The search strategy was developed in stages, beginning with a preliminary search to identify relevant keywords and terms, followed by the application of the search equation across the selected databases.

Table A1, presented in Appendix A, describes the MeSH terms and natural-language terms identified during the development of the search strategy. It also includes alternative subject headings and terms related to the topic, considering the existence of synonymous terminology in the scientific literature [31].

All four databases were accessed through the EBSCOhost interface. The search strategy was developed by combining the selected free-text terms with the Boolean operators AND and OR and was applied consistently across all four selected databases. No database-specific controlled-vocabulary headings or field codes were explicitly incorporated into the final search syntax, and the asterisk (*) was used as the truncation operator. The following search equation was applied: [(Simulation OR Interactive Learning OR Debriefing) AND (Soft Skill* OR Non-Technical Skill* OR Clinical Competenc* OR Clinical Skill* OR Communication OR Decision Making OR Leadership OR Influentials OR Time Management OR Awareness*) AND (Patient Care Team OR Multidisciplinary Care Team OR Healthcare Team OR Medical Care Team OR Interdisciplinary Health Team OR Cooperative Behavior OR Compliant Behavior) AND (Resuscitation* OR Cardiopulmonary Resuscitation OR Heart Arrest OR Cardiac Arrest OR Cardiac Massage OR Heart Massage OR Cardiopulmonary Arrest OR Cardiac Life Support OR Asystole)].

### 2.4. Screening and Selection of Sources of Evidence

The study selection process is represented in the PRISMA flow diagram presented in Appendix B. The database search initially identified 138 articles, of which 80 were retrieved from MEDLINE, 35 from CINAHL, 7 from the Nursing & Allied Health Collection: Comprehensive, and 16 from the Cochrane Central Register of Controlled Trials. Following the removal of 25 duplicate articles before screening, 113 records remained eligible for initial screening.

Subsequently, 51 articles were excluded after title and abstract screening. Of the 62 reports selected for full-text review, 35 were excluded because they did not meet the defined eligibility criteria, namely because they were published in languages other than English, Portuguese, or Spanish (*n* = 6), did not address CPR situations (*n* = 16), did not mention NTS (*n* = 2), did not include simulation settings (*n* = 3), included undergraduate students in their samples (*n* = 6), did not report an eligible participant sample (*n* = 1), or constituted a secondary source that did not report original data (*n* = 1). The final sample comprised 27 sources of evidence.

Study selection was conducted independently by two reviewers, both members of the research team. Any disagreements regarding study eligibility were resolved through discussion with a third reviewer until consensus was reached.

### 2.5. Data Charting Process

Following completion of the study selection process, data were charted using a standardised data extraction table developed by the review team before data charting, in accordance with the JBI recommendations [32]. This table was designed to ensure the consistent and systematic collection, organisation, and recording of relevant information from all included sources of evidence.

Table 1 presents the variables extracted from each included source of evidence, including the authors, year of publication, country of origin, objective, methodology, participants or evidence base, intervention or focus of the source, and key findings.

Data charting was performed independently by two reviewers, both members of the research team. Any discrepancies were resolved through discussion and, when necessary, by consultation with a third and fourth reviewer until consensus was reached.

No contact with the authors of the included sources of evidence was required to obtain or verify additional information.

### 2.6. Critical Appraisal

A critical appraisal of the included sources of evidence was not undertaken, as the primary aim of this scoping review was to map and characterise the available evidence in accordance with the JBI methodology for scoping reviews. Consequently, the sources were not formally weighted or ranked according to their methodological quality.

### 2.7. Data Analysis and Synthesis

The data extracted and recorded in Table 1 were synthesised narratively in accordance with the review objectives and research question, focusing on the relevant concepts, intervention characteristics and outcomes. The sources were compared to identify patterns, similarities and differences across the available evidence.

The sources were considered according to their respective designs and purposes. Findings from controlled trials were analysed in relation to the changes reported following simulation-based interventions; quality-improvement projects were analysed in terms of their implementation, clinical applicability, and changes in team or organisational processes; and technology-development sources of evidence were analysed in terms of how the tools developed supported the conduct of simulation and performance assessment.

The relevant findings from the included primary source types were subsequently integrated into the thematic categories of the review, enabling evidence from different methodological designs to be brought together while preserving the distinctions and similarities between their respective purposes.

NTS were coded deductively according to categories identified in the literature and predefined in the review framework: communication, leadership, teamwork, situational awareness, decision-making, and time management. During data charting, the terminology used to describe NTS and the corresponding outcomes was recorded as reported by the authors of the included sources of evidence. During data synthesis, four members of the review team analysed the relevant outcomes from each included source of evidence and assigned them to the corresponding categories based on their conceptual meaning and the behaviours or performance indicators described, rather than solely on the terminology used by the original authors. When an outcome represented more than one NTS, it was assigned to all relevant categories. The coding process was conducted by four members of the review team and subsequently reviewed and validated by all six members. Any uncertainties or differences in category assignment were discussed by the full review team until consensus was reached.

Simulation modalities and complementary educational interventions were charted and grouped descriptively according to the terminology and intervention characteristics reported by the authors of the included sources of evidence. These elements were analysed separately from the coding of NTS outcomes.

For descriptive mapping, a source was classified as reporting implemented debriefing when it explicitly stated, in the methods or intervention description, that a debriefing session was conducted as part of the simulation or training procedure. References to debriefing that were exclusively contextual or conceptual, without reported implementation, were not counted as instances of debriefing. Where reported, the format and characteristics of the debriefing approach were also charted.

## 3. Results

**Figure A1:**
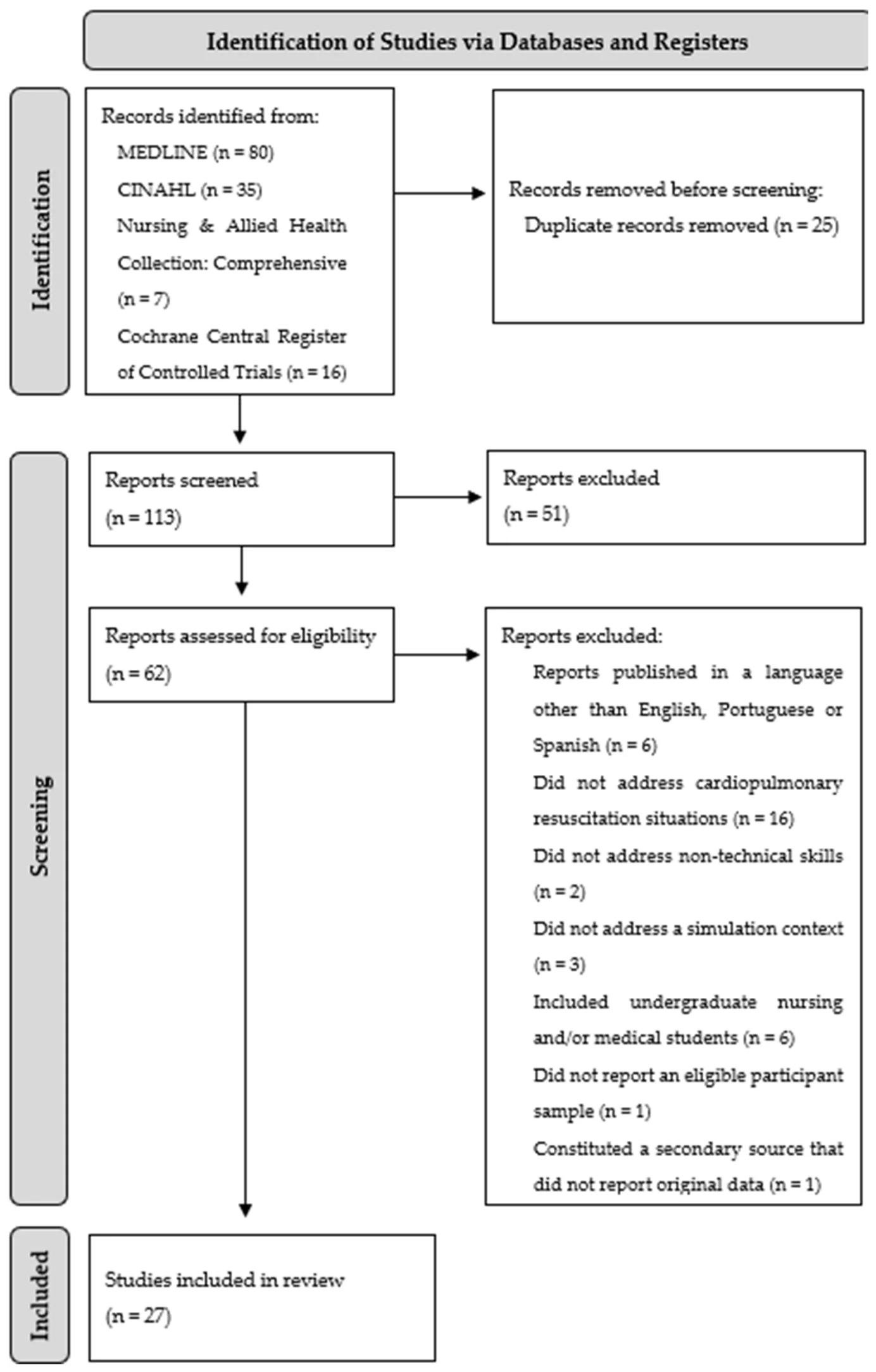
PRISMA flow diagram.

The selection process resulted in the inclusion of 27 sources of evidence in this scoping review. Figure A1 (Appendix B) presents the PRISMA flow diagram, summarising the screening and eligibility process, including the number of records identified, screened, excluded, and included, together with the reasons for exclusion at each stage.

### 3.1. Characteristics of the Sources of Evidence

Of the twenty-seven sources of evidence included in this scoping review, seven were conducted exclusively in the USA [37,46,47,51,52,55,58], six exclusively in Canada [33,39,44,49,50,53], and one jointly in the USA and Canada [36]. The remaining sources were conducted in Austria (*n* = 1) [48], Australia (*n* = 1) [41], Brazil (*n* = 1) [38], Finland (*n* = 1) [54], Germany (*n* = 1) [43], Japan (*n* = 1) [45], South Korea (*n* = 2) [34,59], Switzerland (*n* = 1) [40], Taiwan (*n* = 2) [35,57], and the United Kingdom (*n* = 2) [42,56].

Overall, the sources of evidence analysed converge in their aim of assessing the contribution of clinical simulation to the development of NTS in healthcare teams, particularly communication, leadership, teamwork, SA, and decision-making [34,35,38,39,40,41,42,43,44,46,50,51,54,55,56,57,58]. Some sources of evidence had more specific objectives, namely the development and use of technological tools to support debriefing and SA [33,47], the comparison of different debriefing formats [36,52], or the evaluation of the implementation of structured programmes, such as Team Strategies and Tools to Enhance Performance and Patient Safety (TeamSTEPPS) and the Cognitive Aids for Role Definition (CARD) protocol [37,45,53,55].

Regarding methodological design, quantitative studies predominated, particularly quasi-experimental and experimental studies [34,35,37,43,45,46,53,54,55,59], frequently using validated scales, structured questionnaires, and standardised assessment instruments. In parallel, qualitative, intervention [48], and mixed-methods studies [33,41,42,52] were identified, enabling an understanding of participants’ experiences and perceptions.

Of the sources reporting participant data, interprofessional teams composed of physicians, nurses, and respiratory therapists predominated, reflecting the multidisciplinary nature of the response to CPA. Sample sizes varied considerably, ranging from small groups of eight participants [41] to larger samples comprising 387 professionals [36].

### 3.2. Synthesis of Results

The qualitative analysis of the sources of evidence identified three main thematic categories: (1) clinical simulation as a strategy for skill development; (2) NTS in the context of CPR; and (3) complementary educational interventions. These categories were not predefined but emerged from recurring patterns identified across the charted evidence. Together, they address the review objective and question by mapping how clinical simulation was used to support skill development, which NTS domains and associated outcomes were reported, and which complementary educational interventions were described in the context of CPR.

(1) Clinical Simulation as a Strategy for Skill Development

The included sources of evidence suggest that simulation may support skill development in the context of CPR by providing safe and controlled environments for training, reflection, and the consolidation of learning.

Different simulation modalities were identified. Several sources used high-fidelity simulation involving advanced manikins and/or highly realistic scenarios [33,36,37,44,56], while one source used immersive virtual reality-based cardiac arrest simulations [52]. Low-fidelity in situ simulation was also reported [57]. However, no included source directly compared virtual reality-based simulation with advanced manikin-based simulation; therefore, differences in NTS outcomes between these modalities could not be determined.

In situ simulation emerged as the most frequently used approach [43,45,47,48,51,54,57] and was described as enabling training in real clinical settings, supporting contextualised learning and facilitating the identification of organisational weaknesses with potential implications for patient safety.

Additionally, several sources of evidence emphasised the importance of the frequency and repetition of simulation-based training for the retention and consolidation of the skills developed.

Despite the generally favourable findings, some sources of evidence reported limited effects or a decline in the effects of the interventions over time. Specifically, the level of physical realism of the simulators had no relevant impact on the measured outcomes [36]. Moreover, although improvements in individual and team performance were observed following simulation, these effects diminished over time [35]. Consistent with this finding, another study reported only partial retention of NTS following the initial training, reinforcing the importance of regular training and periodic educational programmes for their long-term maintenance [52].

(2) Non-Technical Skills in the Context of Cardiopulmonary Resuscitation

NTS emerged as relevant elements in supporting team response during CPR. Across the included sources of evidence, communication was the most frequently reported skill and was described as relevant for team coordination, the sharing of critical information, and reduction in errors in high-pressure settings [33,34,37,38,39,41,44,45,46,47,50,52,55,56,57,58]. At the review level, communication, leadership, and teamwork were also identified as relevant dimensions of simulation-based education [34]; however, these findings were interpreted as contextual evidence rather than as additional intervention-level findings.

Leadership was also frequently identified as relevant to the organisation of the response, priority setting and collective team performance. Some sources reported associations between leaders’ experience, confidence, and leadership profile and intervention-related outcomes [37,38,40,51,52,54,57].

Teamwork was associated with role clarity, group cohesion, and the ability to respond in a coordinated manner to complex scenarios [34,35,38,40,41,48,50]. In parallel, SA was identified as an essential skill for monitoring the clinical environment, anticipating needs and supporting timely decision-making [33,50,55,56,57,59].

Other relevant skills identified included decision-making, interprofessional coordination, and self-confidence. The latter was associated with greater assurance and improved performance during emergency situations [43,47,48].

However, the effects of the interventions on the different NTS were not consistent across sources of evidence. A single, stand-alone educational session on NTS did not produce statistically significant improvements in the skills assessed, as no significant differences were identified between the intervention and control groups [54]. Similarly, integrating structured communication training into a simulation-based intervention did not result in significant differences between the groups in communication clarity [59]. Regarding in situ simulation, despite the improvements observed in overall team performance and knowledge of neonatal resuscitation guidelines, no overall improvement in NTS was identified, although participants’ baseline performance in these skills was already high [48]. Likewise, the long-term implementation of low-fidelity in situ simulation did not result in consistent improvements in crisis resource management skills [57]. In addition to the variability in intervention effects, weaknesses were identified in specific skills, with leadership and decision-making showing the lowest levels of performance [56]. Team leaders were also found to be less reliable than other team members in assessing overall team performance, which may have been related to the greater cognitive workload experienced by leaders during resuscitation [40]. Finally, the interpretation and comparison of findings relating to NTS are also limited by the lack of standardisation among the assessment instruments currently available [42].

(3) Educational Interventions Complementary to Simulation

In addition to simulation itself, the sources of evidence highlight the relevance of several complementary educational interventions in the development of NTS.

Across the 27 included sources of evidence, debriefing was the most frequently addressed complementary educational strategy and was reported in 21 sources (77.8%) [33,34,35,36,37,38,39,40,41,43,44,45,46,47,48,50,51,54,55,56,57]. However, its role varied according to source type. Twenty-one sources explicitly reported that a debriefing session was conducted as part of the simulation or training procedure [33,34,35,36,37,38,39,40,41,43,44,45,46,47,48,50,51,54,55,56,57]. Reported formats included structured facilitator-led debriefing, scripted and non-scripted debriefing, video-assisted debriefing, and structured group debriefing [35,36,39,43]. One source comparing scripted and non-scripted debriefing delivered by novice instructors reported greater improvements in knowledge and team leader behavioural performance when a standardised script was used. However, no significant difference was observed in overall clinical team performance [36].

Several sources of evidence also reported the use of supporting technologies, such as real-time feedback systems, digital devices for monitoring SA (SA Display), enabling a more objective assessment of dimensions such as communication, leadership, and team coordination [33,45].

Other complementary strategies were also identified, namely e-learning, preparatory workshops, and the use of performance videos. These were used to stimulate critical thinking, promote prior preparation and facilitate self-assessment and critical reflection processes [34,35,42,43,50].

However, limitations and gaps in the evidence relating to educational interventions complementary to simulation were also identified. Although the SA Display was considered useful in supporting situational awareness and the organisation of clinical information, participants expressed concerns regarding potential overreliance on the device, the possibility of outdated information being displayed and reduced verbal communication among team members [33]. Another interactive visual system was found to indirectly support the development and reinforcement of communication, leadership, and situational awareness; nevertheless, the need to investigate its direct impact in future studies was emphasised [58].

In summary, the included sources suggest that clinical simulation, particularly when combined with structured debriefing and complementary educational technologies, may support the development of NTS among healthcare teams involved in CPR. Reported improvements in communication, leadership, teamwork, and situational awareness may be relevant to team performance and the quality of the response during CPR. However, the findings were not consistent across sources; some interventions produced no significant improvements in specific NTS, the improvements observed diminished or were only partially retained over time, and some complementary technologies raised concerns among participants or had not yet been formally evaluated or validated. Given the heterogeneity of the included sources and the absence of a formal critical appraisal, no causal conclusions can be drawn regarding the effectiveness of simulation or its impact on patient safety and clinical outcomes.

## 4. Discussion

The findings of this scoping review provide an overview of how clinical simulation may contribute to the development of NTS in the context of CPR, particularly communication, leadership, teamwork, SA, and decision-making. Three thematic categories emerged from the analysis of the evidence, providing insight into the reported outcomes, educational strategies, and factors influencing the use of simulation for team training in critical care settings.

Compared with recent evidence syntheses focusing on undergraduate medical education or on simulation-based NTS development across interprofessional healthcare teams in multiple clinical fields [21,22], the present review provides a context-specific map centred on qualified healthcare teams involved in CPR. Its added value lies in integrating, within a single CPR-specific synthesis, evidence on simulation modalities, NTS domains, complementary educational components, assessment approaches, and reported outcomes. This mapping brings together favourable, null, and inconsistent findings and highlights the decline or partial retention of some reported improvements, as well as the preliminary status of several technology-based interventions. By making visible variation in study designs, NTS concepts, and assessment instruments, the review also clarifies the methodological constraints that limit comparability and prevent firm conclusions regarding effectiveness, transfer to actual resuscitation practice, and patient outcomes. Taken together, these findings may inform healthcare professionals, educators and healthcare organisations involved in designing and evaluating simulation-based training programmes for CPR.

(1) Clinical Simulation as a Strategy for Skill Development

The included sources indicate that clinical simulation has been used as an educational strategy for developing NTS in the context of CPR. Several sources reported improvements in team performance and related outcomes. However, given the methodological and conceptual heterogeneity of the included evidence and the descriptive mapping purpose of this scoping review, these findings do not permit firm conclusions regarding the effectiveness of simulation or its direct effects on patient safety and the quality of the response in critical situations [52,55,59,60,61].

Among the different modalities identified, in situ simulation emerged as one of the most frequently reported approaches and was described as enabling training within real clinical environments and supporting contextualised learning. In addition to its use in technical and relational skill training, this modality may facilitate the identification of latent system failures, allowing risks to be anticipated and opportunities for improving organisational processes to be identified before adverse events occur [62]. The findings also suggest that in situ training programmes may support interprofessional coordination, role clarification, and team effectiveness in high-pressure situations [48].

The sources of evidence analysed also reported the use of hybrid models, or blended learning models combining face-to-face teaching, clinical simulation, and virtual or digital components. This approach was described as potentially facilitating the integration of theoretical knowledge, TS, and NTS, while providing more flexible learning experiences adapted to professionals’ needs [62].

Current evidence further suggests the relevance of clearly defined learning objectives, appropriate scenario design and the facilitator’s active role in supporting meaningful learning [52,61,62,63]. These findings are consistent with Jeffries’ Simulation Model, which conceptualises simulation as an active learning experience structured around interdependent components, including the context, simulation design, facilitator, participants and expected outcomes [10,15,16].

However, the findings do not support a uniform view of simulation outcomes. The absence of a relevant impact of simulator physical realism on the measured outcomes suggests that technological sophistication alone may not determine these outcomes [36]. When considered alongside the available literature, this finding indicates the potential importance of intentional and appropriately structured instructional design aligned with the intended learning objectives [61,62,63,64]. This interpretation is consistent with evidence suggesting that physical resemblance should be considered in relation to functional task alignment, learner engagement, and transfer of learning rather than as an independent determinant of educational value [65]. From a practical perspective, this may be particularly relevant in resource-limited settings, where access to high-fidelity simulators may be restricted. In such contexts, available resources may be prioritised towards scenario design, facilitator preparation, structured debriefing, and repeated practice [61,62,63,64,65]. Recent evidence from nursing education has also identified low-cost simulation as a potential means of addressing both technical and non-technical competencies, while highlighting inconsistent cost reporting and a lack of standardisation [66]. Lower-fidelity approaches may therefore still support NTS development when these educational components are carefully implemented [36,65], although the available evidence does not establish equivalence between fidelity levels. Accordingly, simulation outcomes may depend not only on the technology employed but also on the educational design and its capacity to promote critical reflection and the transfer of learning to clinical practice.

With regard to specific simulation modalities, the included evidence encompassed both immersive virtual reality-based simulation and advanced manikin-based simulation; however, no source directly compared these modalities using the same NTS outcomes. The virtual reality-based source compared cognitive and communication processes between expert and novice team leaders rather than simulation formats [52], whereas the manikin-based sources differed substantially in their designs, participants, scenarios and outcome measures [33,36,37,44,56]. Consequently, it was not possible to determine whether statistically significant differences exist between immersive virtual reality and advanced manikin-based simulation. This limitation is consistent with the broader literature. A systematic review focusing on virtual reality in CPR training concluded that this field remains at an early stage and that further research using standardised approaches is required to identify the most appropriate training modality [67]. Similarly, a broader systematic review comparing virtual reality with traditional simulation found insufficient evidence to support the superiority of either approach [68]. Head-to-head studies using standardised and validated NTS measures are needed to establish whether particular modalities offer advantages for specific learning objectives, professional groups or clinical contexts.

Furthermore, the reported decline in improvements over time and the partial retention of NTS suggest that the initial acquisition of these skills does not guarantee their maintenance [35,38]. These findings indicate the potential value of repeated practice, learning distributed over time and periodic reinforcement, rather than reliance on isolated training sessions, particularly when medium- and long-term retention of NTS is intended [61,63,64,69]. The frequency, continuity and integration of simulation into training programmes may therefore be relevant to supporting the retention of reported improvements.

(2) Non-Technical Skills in the Context of Cardiopulmonary Resuscitation

NTS emerged as relevant elements in supporting team responses during CPR. Across the included sources of evidence, communication was the most frequently reported skill and was described as relevant to team coordination, the sharing of critical information, and the prevention of errors in high-pressure settings [33,34,37,38,39,41,44,45,46,47,50,52,55,56,57,58]. Structured communication strategies, namely closed-loop communication, were associated with reported improvements in the clarity of instructions, task coordination and the reduction in conflict in critical situations [63,70]. These findings highlight the potential relevance of effective communication to patient safety and coordination of resuscitation teams.

The included evidence encompassed both paediatric/neonatal and adult or predominantly adult CPR settings. In paediatric and neonatal contexts, NTS were examined within specialised interprofessional neonatal, paediatric critical care and trauma teams, with particular attention to communication, teamwork, leadership, and SA [34,36,37,38,39,44,46,47,48,50,54,55,58]. In adult or predominantly adult settings, similar NTS were examined across emergency, perioperative, surgical, trauma and ward-based teams, including role allocation, crisis resource management and decision-making [41,42,43,45,51,53,56,57,59]. The cognitive demands placed on team leaders were specifically considered in studies addressing self-monitoring and leadership processes [40,52]. Differences in team composition and clinical organisation may influence how these skills are enacted; however, no included source directly compared paediatric/neonatal and adult CPR settings, and cognitive load was not assessed consistently across these contexts. The findings should therefore be interpreted within the clinical and organisational characteristics of each population, without direct extrapolation between age groups.

Interprofessional teamwork also emerged as a central dimension in the included sources of evidence. The joint participation of different professionals in simulated scenarios was associated with improvements in collaboration, coordination and group cohesion, which may support more coordinated responses in highly complex settings [38,55,71]. These findings suggest the potential value of simulation in supporting collaborative practices and a culture of safety.

In parallel, SA and decision-making were identified as essential skills in the context of CPR, supporting continuous monitoring of the clinical environment, anticipation of needs and timely response to changes in the condition of the critically ill patient. The evidence analysed suggest that simulation may support the development of these skills through repeated exposure to complex scenarios and critical reflection on team performance.

The included sources reported outcomes at individual, team and organisational levels. At the individual level, improvements were reported in participants’ self-confidence, responsiveness and communication skills [48,63,70]. At the team level, sources reported improvements in coordination, the flow of interventions and team effectiveness [38,71]. At the organisational level, in situ simulation was described as having the potential to facilitate the identification of systemic weaknesses and opportunities for improving the safety and organisation of care [60,72].

Despite the generally favourable outcomes reported, some sources of evidence found no significant or consistent improvements in NTS, indicating that responses to the interventions varied across skills, contexts, and participants [48,54,57,59]. This variability may reflect differences in programme structure and duration, participants’ previous exposure to training and their baseline levels of performance [61,63]. In one study, no overall improvement in NTS was observed in a sample whose baseline performance was already high, potentially limiting the possibility of detecting additional improvements [48]. The absence of statistically significant differences should therefore not be interpreted in isolation as evidence that an intervention was ineffective, particularly when high baseline performance reduces the possibility of detecting further improvement.

This variability was particularly evident in relation to leadership, which was identified as a key skill associated with collective performance. Recent evidence suggest that more experienced leaders may have a greater capacity to anticipate events, potentially facilitating the prioritisation of interventions, team management and decision-making in complex situations [52,73,74]. In contrast, less experienced leaders were reported to focus on the completion of immediate tasks, sometimes compromising their overall understanding of the clinical situation [74]. The findings further suggest that simulation may support the development of shared mental models within the team, with potential benefits for SA, coordination and collective decision-making [52,74,75]. Nevertheless, leadership and decision-making appeared to be particularly demanding, having shown the lowest levels of performance in one of the included sources of evidence [56]. Taken together, these findings are consistent with evidence identifying leadership experience and behavioural leadership strategies as factors associated with team performance during CPR [73].

Recent qualitative evidence from trauma settings further suggests that leadership development may be influenced by clinical experience, the challenge of simultaneously assuming the roles of leader and learner, and informal learning opportunities that are not always standardised. Collectively, these findings suggest the potential value of integrating leadership development into structured, progressive and contextually relevant training programmes, rather than relying exclusively on isolated interventions [76].

In addition to the challenges associated with leadership performance, limitations were identified in team leaders’ ability to assess overall team performance. Team leaders were reported to provide less reliable assessments than other team members, which may have been related to the greater cognitive workload experienced during resuscitation [40]. A similar pattern was reported in another high-fidelity clinical simulation context, in which ratings provided by team leaders and team members were initially higher than those assigned by independent observers. However, these differences were no longer significant following repeated exposure to the scenarios, suggesting that repeated practice and external feedback may contribute to a more accurate appraisal of performance [77]. Caution is therefore warranted when self-assessment is used as the sole method of monitoring performance, and its combination with structured external assessment should be considered.

A further methodological concern relates to the lack of standardisation among instruments used to assess NTS, which limits the comparison of findings and may contribute to the heterogeneity observed across sources of evidence [42]. This concern is consistent with previously reported inconsistencies in the concepts, terminology and measures used in the literature on resuscitation teams [72]. A systematic review also identified substantial variation in the number of domains and items included in the available instruments, together with limited evidence regarding essential aspects of the validity and reliability of most measures [78]. The interpretation of the reported simulation outcomes should therefore consider the robustness and comparability of the assessment instruments employed, reinforcing the need for validated and standardised measures.

(3) Educational Interventions Complementary to Simulation

In addition to simulation itself, the sources of evidence analysed several complementary educational interventions in the development of NTS. Among these, debriefing was frequently reported as an important stage of the educational process, as well as a strategy that may support the consolidation of knowledge, critical reflection and the identification of areas for improvement [61,63,79].

Debriefing was delivered using different approaches across the included sources of evidence, including structured facilitator-led, scripted and video-assisted formats [36,43]. However, the available findings do not allow conclusions to be drawn regarding the relative effectiveness of these different approaches. One source comparing scripted and non-scripted debriefing delivered by novice instructors reported greater improvements in knowledge and team leader behavioural performance when a standardised script was used [36].

The integration of complementary technologies, such as real-time feedback systems, digital tools for monitoring SA and performance analysis software, was also reported to have potential to increase the objectivity of feedback and support reflective processes [79,80,81]. Additionally, strategies such as e-learning, preparatory workshops and the use of performance videos were reported to support participants’ preparation, critical thinking, self-assessment and reflection.

However, the limitations reported in technology-based interventions suggest that these tools may be best regarded as complementary resources rather than substitutes for important interactions among team members. Although the SA Display was perceived as useful in supporting SA and the organisation of clinical information, participants raised concerns regarding potential overreliance on the device, the possibility of outdated information being displayed and reduced verbal communication among team members [33]. These concerns suggest that technological support may introduce new risks when it is not appropriately integrated into clinical workflows or when its use interferes with fundamental team behaviours, particularly communication and the active sharing of information.

This interpretation is consistent recent evidence concerning digital decision-support tools in resuscitation settings, suggesting that their safe use may depend on the timely, accurate and standardised entry of information and the appropriate updating of task status. Incomplete or outdated information may compromise the accuracy of alerts and generate inappropriate recommendations [82].

Furthermore, the available evidence concerning some of these technologies remains preliminary. An interactive visual system was reported to have the potential to indirectly support communication, leadership and SA; however, its direct impact on NTS required further investigation [58]. Similarly, the TeamVis platform was described as having the potential to support debriefing and the analysis of team performance, but its effectiveness had not been formally evaluated or validated [48].

This uncertainty is consistent with a recent systematic review of cognitive aids used during resuscitation, which identified potential benefits for healthcare professionals but emphasised that the available evidence was derived exclusively from simulation studies, ranged from moderate to very low certainty and was characterised by considerable methodological heterogeneity [83]. No evidence from actual resuscitations was identified that would allow the impact of these tools on clinical performance or patient outcomes to be determined.

Taken together, these findings suggest the potential value of multimodal and integrated educational approaches in which simulation is complemented by strategies intended to support reflection, structured feedback and the retention of learning. Nevertheless, caution is required when interpreting the reported benefits of technology-based interventions. Before their widespread adoption, their validity, effectiveness, usability, integration into clinical workflows and effects on team communication and performance should be formally evaluated. Technology may therefore be most appropriately used to support reflection, information sharing and decision-making without replacing active interaction among healthcare professionals or unnecessarily increasing their cognitive workload.

### Limitations of the Evidence, Review Limitations and Implications for Future Research

Important limitations remain in the available evidence. Participant and sample characteristics were not consistently reported across the included sources; several did not provide exact participant numbers or sufficiently detailed demographic and professional characteristics, while one technology-development source did not report a formal study sample [37,43,49,51,53,58]. This incomplete reporting restricts the characterisation of the populations and evidence bases represented and the assessment of the applicability of the reported findings to other professional groups and clinical contexts. More broadly, the methodological heterogeneity of the sources of evidence, the use of assessment instruments that were not always validated and the scarcity of objective clinical indicators hinder comparisons across sources and limit the generalisability of the findings [61]. Variability in the training frequency, facilitators’ experience, simulation formats and outcome measures may also have influenced the observed outcomes [62,73]. Furthermore, the decline in some reported improvements over time and the partial retention of NTS indicate that immediate improvements following simulation should not be assumed to persist in the medium or long term.

The findings should also be interpreted in light of the limitations of the review process. The four databases were selected to provide complementary coverage of biomedical, nursing, allied health and intervention-focused literature, all database searches were performed through a single platform, EBSCOhost. Although MEDLINE is the largest component of PubMed, the two resources are not fully equivalent, as PubMed also includes records from other National Library of Medicine resources and records that are not indexed in MEDLINE [84]. As PubMed was not searched as a separate resource, potentially relevant PubMed-only records may not have been retrieved, which may have limited the comprehensiveness of the review. Reliance on a single search platform may have influenced retrieval because database implementation, field mapping, controlled-vocabulary functions, search syntax and filters can vary across providers and native database interfaces. Consequently, potentially relevant sources of evidence indexed exclusively in other databases, or retrievable through alternative interfaces, may not have been identified, which may have limited the comprehensiveness of the review. Grey literature sources and trial registries were not systematically searched, which may have limited the comprehensiveness of the review and increased the possibility of publication bias through the omission of relevant unpublished or ongoing studies. Although additional grey literature was consulted to support the theoretical background and contextualise the discussion, these sources were not assessed against the eligibility criteria, subjected to the study-selection and data-charting processes, or included as sources of evidence in the review.

The use of the EBSCOhost “Full Text” limiter may have excluded potentially eligible records that were not identified by the platform as having full text available, including articles that might have been obtainable through other retrieval routes. This represents a potential source of selection bias and may have further limited the comprehensiveness of the review.

The methodological heterogeneity of the included sources broadened the scope of the review but also increased the complexity of the synthesis. Differences in study designs, methods, participant groups, interventions, assessment instruments, follow-up periods and reported outcomes limited direct comparability across sources. Accordingly, the findings were integrated for descriptive and thematic mapping, and comparisons across sources should be interpreted with caution.

Furthermore, restricting the sources of evidence to those published in English, Portuguese or Spanish may also have resulted in language bias and the omission of relevant evidence published in other languages. A critical appraisal of the included sources was not undertaken. Although this decision is consistent with scoping review methodology, the methodological quality and risk of bias of the individual sources were not formally assessed. Accordingly, the findings provide an overview of the available evidence and should not be interpreted as pooled estimates of effectiveness or definitive evidence of causal effects.

Future research should therefore employ more robust methodological designs, validated and standardised assessment instruments, and longitudinal evaluations of NTS retention. Further studies are also needed to examine whether improvements observed in simulated settings are transferred to clinical practice and result in measurable effects on actual team performance, patient safety and patient outcomes during CPR. Greater consistency in the definition and measurement of NTS, together with formal evaluation of complementary technologies and debriefing approaches, may improve comparability across studies and strengthen the evidence available to guide educational practice.

## 5. Conclusions

This scoping review mapped the available evidence concerning the use of clinical simulation for the development of NTS among healthcare teams involved in CPR. Overall, the included sources suggest that simulation, particularly when conducted in situ and combined with structured debriefing and complementary educational strategies, may support the development of communication, leadership, teamwork, SA and decision-making. The sources also reported outcomes related to contextualised learning, interprofessional coordination and reflection on team performance, which may have implications for patient safety, team effectiveness and the quality of care in critical settings.

Structured debriefing and complementary educational strategies were frequently reported as components that may support the consolidation of learning and the retention of the skills developed.

However, the reported outcomes were not consistent across all sources of evidence, skills or participants, and some reported improvements diminished or were only partially retained over time. Evidence concerning certain complementary technologies also remained preliminary, with some tools not yet formally evaluated or validated for effectiveness.

These conclusions should be interpreted in light of the methodological and conceptual heterogeneity of the included sources of evidence and the limited availability of objective clinical outcomes. Consistent with the descriptive mapping purpose of this scoping review, the included sources were not formally appraised or weighted according to their methodological quality. Furthermore, restricting the search to four databases, not systematically searching grey literature and trial registries, and applying language restrictions may also have resulted in the omission of relevant evidence. Accordingly, these findings should be interpreted as a descriptive mapping of the available evidence and do not establish the effectiveness of simulation or its direct effects on patient safety and clinical outcomes.

Within these limitations, the included sources suggest that clinical simulation may be a relevant component of NTS training in CPR. However, the available evidence does not allow conclusions regarding the comparative effectiveness of different simulation modalities, fidelity levels or educational components. Further methodologically robust quantitative and longitudinal studies, using validated and standardised outcome measures, are needed to confirm the reported trends and determine whether improvements are sustained and transferred to clinical practice, and whether they are associated with measurable changes in actual team performance, patient-safety indicators and patient outcomes.

## Figures and Tables

**Table 1:** Characteristics of the sources of evidence included in the scoping review.

Author(s) (Country/Year) [Reference]	Objective	Methodology	Participants or Evidence Base	Key Findings	
				Intervention or Focus of the Source	Conclusions
Calder et al. (Canada, 2018) [33]	To explore healthcare professionals’ perceptions of the usefulness of the Situational Awareness (SA) Display in supporting communication and SA during simulated cardiopulmonary resuscitation (CPR).	Qualitative study using focus groups, high-fidelity simulation and qualitative data analysis.	33 emergency department healthcare professionals: 20 participated in focus groups and 13 in simulation and debriefing sessions, organised into teams of 4–5 members.	Development and evaluation of a bedside SA Display across six high-fidelity simulated CPR scenarios (three with and three without the display), followed by structured debriefing after each scenario.	Participants perceived the display as useful for supporting SA, reducing cognitive workload, and organising clinical information, with less redundant communication. Concerns included potential overreliance on the display, outdated information, and reduced verbal communication.
Chae & Shon (Republic of Korea, 2024) [34]	To develop, implement, and evaluate an interprofessional education programme combining simulation and e-learning to support teamwork, communication, clinical performance, clinical judgement, and interprofessional attitudes.	Pre-experimental pre-test/post-test study delivered over three blended-learning sessions.	27 healthcare professionals: 9 physicians and 18 nurses.	Development of an educational programme combining online learning, structured communication training and simulation-based crisis scenarios, including CPR. Each session concluded with structured debriefing.	Improvements were reported following the programme in teamwork, communication, clinical performance, clinical judgement and interprofessional attitudes. The blended-learning and structured communication components were also described as supporting critical thinking and reflective practice.
Chen et al. (Taiwan, 2025) [35]	To analyse teamwork competencies across levels of clinical experience and compare learning retention following simulation with or without an additional workshop.	Quasi-experimental comparative study involving two intervention groups in a hospital setting.	89 resident physicians: 51 received simulation and structured debriefing, and 38 participated in an additional educational workshop.	Simulated CPR sessions at months 1, 6 and 11, each comprising a 12 min scenario followed by a 10 min expert-led structured debriefing; one group also received an additional workshop.	Improvements in individual performance and teamwork were reported following simulation, but diminished over time, indicating limited retention and the need to investigate more sustainable reinforcement strategies.
Cheng et al. (USA and Canada, 2013) [36]	To assess the effects of scripted debriefing and simulator physical realism on knowledge and team performance during simulated cardiopulmonary arrest (CPA).	Randomised clinical trial with a factorial pre-test/post-test design.	387 healthcare professionals organised into 90 interprofessional teams; debriefing was delivered by 90 instructors with little or no previous experience.	Twenty-minute simulation and debriefing sessions comparing scripted with non-scripted debriefing and simulators with high versus low physical realism.	Scripted debriefing was associated with greater improvements in knowledge and team leader behavioural performance, but no significant difference was observed in overall clinical team performance. Simulator physical realism had no relevant effect on the measured outcomes.
Figueroa et al. (USA, 2013) [37]	To introduce Team Strategies and Tools to Enhance Performance and Patient Safety (TeamSTEPPS) during interprofessional simulation-based training and evaluate its effects on perceptions of teamwork, confidence and communication.	Quasi-experimental study without a control group, with assessments conducted before the intervention, immediately afterwards and three months later, using quantitative questionnaires.	37 healthcare professionals: 5 resident physicians, 5 respiratory therapists, and 23 nurses; the professional category of 4 participants was not reported.	Theoretical training on TeamSTEPPS and the CPR algorithm, followed by six high-fidelity simulations based on real crisis scenarios. Each session concluded with structured debriefing focused on leadership and communication.	Improvements in communication, confidence and team performance were reported following simulation, supporting the application of CPR algorithms. Leadership and communication skills were retained at three months. TeamSTEPPS was considered effective in the paediatric setting, although further studies using objective measures were recommended.
Friedlaender et al. (Brazil, 2025) [38]	To evaluate the acquisition and retention of non-technical skills (NTS) among healthcare professionals participating in a simulated paediatric CPR scenario.	Prospective cohort study conducted at the simulation centre of a paediatric hospital.	85 paediatric resident physicians and nurses participated initially, organised into 16 interprofessional teams; 58 completed all study phases.	Each team completed a simulated paediatric CPR scenario twice on the same day and again approximately 107 and 161 days later to assess NTS retention. Scenarios focused on leadership, communication, teamwork and decision-making and were followed by structured debriefing.	Improvements in NTS were reported following simulation-based training, with partial retention observed over time. The authors highlighted the importance of regular simulation and periodic educational programmes to support the long-term maintenance of these competencies.
Gilfoyle et al. (Canada, 2007) [39]	To develop and evaluate an educational workshop designed to promote the acquisition and retention of leadership skills in advanced paediatric resuscitation.	Educational intervention study.	20 paediatric residents: 15 attended the initial workshop and 14 completed the six-month follow-up.	Workshop combining introductory training on leadership and team dynamics with two simulated CPR scenarios, each followed by structured debriefing. A follow-up workshop at six months included repetition of one of the initial scenarios to assess competency retention.	Simulation provided a safe environment for experiential learning without compromising patient safety. The intervention targeted key leadership competencies, including task allocation, recognition and management of team limitations, continuous reassessment of team performance to minimise fixation errors, and effective communication through a calm tone of voice, clear instructions, and closed-loop communication.
Hautz et al. (Switzerland, 2020) [40]	To compare interprofessional teams’ self-assessments of their performance during simulated CPR with objective external assessments of team performance.	Quantitative observational study conducted in an emergency department.	115 healthcare professionals: 22 physician team leaders and 93 team members, predominantly nurses. Teams comprised 3–6 members, including at least one physician and two nurses.	Simulated CPR scenarios followed by self-assessment of perceived performance and structured debriefing focused on leadership and teamwork competencies.	Team members’ self-assessments of collective performance were consistent with objective external evaluations and reflected an accurate perception of teamwork quality. In contrast, team leaders were less reliable in assessing overall team performance, which the authors suggested may be related to the increased cognitive workload experienced by leaders during resuscitation, potentially affecting their perception of team performance.
Hibberson et al. (Australia, 2023) [41]	To explore Australian multidisciplinary perioperative team members’ experiences of simulation training, including barriers and enablers, training frequency, scenarios used and perceived changes in teamwork and communication.	Qualitative descriptive exploratory study based on individual semi-structured interviews and thematic analysis.	8 healthcare professionals: 1 anaesthetist and 7 nurses.	No intervention was implemented by the authors. Participants described previous multidisciplinary simulation training in Australian perioperative units, most commonly involving basic and advanced life support, with cardiac advanced life support and other arrest-related scenarios also reported. Post-simulation debriefing was also described.	Participants perceived simulation as providing a safe environment for practising emergency management. Teamwork was the only NTS explicitly reported as improved, particularly team cohesion, understanding of professional roles and confidence in other team members. Findings were derived from different perioperative emergency simulations and were not disaggregated by scenario type.
Higham et al. (UK, 2023) [42]	To evaluate the validity and usability of three assessment tools designed to measure NTS during simulated CPR scenarios.	Mixed-methods observational study involving the secondary analysis of video recordings of simulated CPR scenarios.	10 CPR simulation videos independently assessed by three experienced consultant anaesthetists with expertise in simulation-based education, yielding a total of 30 assessments for each instrument.	Previously recorded clinical simulation videos were retrospectively evaluated using three NTS assessment tools. To ensure methodological consistency, video review was standardised through independent and randomised assessment by the evaluators.	The authors reported that the assessment of NTS during simulated CPR is feasible; however, currently available assessment tools lack standardisation. The authors emphasise the need to develop simpler, more specific and universally applicable instruments for the assessment of NTS.
Knobel et al. (Germany, 2018) [43]	To evaluate the impact of an in situ simulation programme with video-assisted debriefing on trauma resuscitation effectiveness and healthcare professionals’ self-confidence.	Retrospective study conducted at a trauma centre using questionnaire-based data collection.	Approximately 190 healthcare professionals, including 17 trauma surgeons, 60 anaesthesiologists, approximately 80 nurses and 10 radiology technologists.	Simulation sessions were conducted monthly using authentic clinical crisis scenarios, including CPR. Each session concluded with video-assisted debriefing, enabling a detailed review of the different phases of the resuscitation process.	The authors reported a significant improvement in healthcare professionals’ self-confidence and perceived preparedness following participation in the simulation programme. The authors emphasise the importance of ongoing simulation training in promoting the acquisition and long-term retention of NTS.
Kotsakis et al. (Canada, 2015) [44]	To equip healthcare professionals with the knowledge and skills to recognise and manage acute clinical deterioration in children and function effectively within multidisciplinary teams, with emphasis on basic life support, structured communication and crisis resource management.	Descriptive educational study outlining the development and implementation of a Paediatric Acute Care Course, followed by an evaluation of participants’ perceptions and learning outcomes.	89 healthcare professionals: 38 paediatric physicians and 51 nurses.	Paediatric Acute Care Course delivered across four courses over two and a half years using high-fidelity simulation. Each team completed six scenarios, each followed by a 20 min structured debriefing analysing individual and team behaviours.	Participants reported increased confidence, improved communication and better understanding of team roles and teamwork. Simulation was described as providing interprofessional teams with opportunities to undertake different roles and develop a deeper understanding of individual responsibilities within the resuscitation team. Greater role clarity enabled participants to better anticipate team members’ actions and clinical needs, contributing to more efficient resuscitation performance.
Laco & Stuart (Japan, 2022) [45]	To develop and implement a simulation-based training programme to improve CPR skills and teamwork among healthcare professionals at a military base.	Quality improvement project with a quasi-experimental design, with data collected before and after the intervention using direct observation instruments and questionnaires, without a control group.	Participants 18 healthcare professionals: 5 nurses, 5 physicians and 8 medical technicians.	In situ CPR simulation incorporating emergency scenarios, real-time manikin-based feedback, structured briefing and debriefing, and performance assessment using checklists and scales, including TeamSTEPPS.	Participants reported improved perceptions of NTS, with significant improvements in communication, teamwork and team structure according to TeamSTEPPS. Structured briefing, feedback and debriefing were highlighted as important components supporting these perceived improvements. The authors concluded that high-fidelity simulation was effective in developing NTS.
Laurendine et al. (USA, 2020) [46]	To assess teams’ perceptions of the skills developed, as well as the impact of simulation and debriefing in a neonatal intensive care unit.	Quality improvement study using mixed questionnaires and descriptive and categorical analysis.	83 healthcare professionals: 60 registered nurses, 15 respiratory therapists, 4 neonatologists, 3 neonatal nurse practitioners and 1 physician assistant.	Simulation sessions were conducted at varying intervals, followed by structured debriefing.	The authors reported that simulation and debriefing improved communication, confidence and organisation within the team. The authors also emphasised the need for regular NTS training.
McLaughlin et al. (USA, 2018) [47]	To evaluate the impact of an in situ simulation-based multidisciplinary training programme on healthcare professionals’ self-confidence within a paediatric trauma resuscitation team.	Prospective observational cohort study conducted over a two-year period.	A total of 190 healthcare professionals were eligible to participate. Survey responses were obtained from 93 participants at baseline, 42 at one month and 150 at the two-year follow-up. Participants included physicians, nurses, radiographers, pharmacists and respiratory therapists.	A multidisciplinary training programme based on in situ simulation was implemented using authentic clinical crisis scenarios, including CPR, followed by structured debriefing.	The authors reported that the in situ simulation programme significantly increased healthcare professionals’ self-confidence. Furthermore, structured debriefing played a pivotal role in reinforcing learning and promoting the long-term retention of knowledge and skills. Simulation also facilitated the identification of communication and logistical shortcomings within the team, highlighting areas requiring further training and quality improvement.
Mileder et al. (Austria, 2024) [48]	To evaluate the impact of in situ simulation on the quality of neonatal stabilisation during CPR.	Quality improvement study conducted in a Level IV Neonatal Intensive Care Unit.	48 healthcare professionals: 21 physicians and 27 nurses, organised into interprofessional teams.	In situ simulation sessions were conducted over a four-month period. Each session lasted 30–50 min and included a structured debriefing.	In situ simulation significantly improved team performance and participants’ knowledge of neonatal resuscitation guidelines. However, no overall improvement in NTS was observed, with baseline NTS performance already high before the intervention. The findings suggest that in situ simulation may be a promising strategy for strengthening teamwork in neonatal resuscitation.
Nagji & Sadiq (Canada, 2012) [49]	To describe the implementation of rural emergency simulation sessions designed to prepare interdisciplinary teams to manage CPA.	Descriptive and reflective report of an educational initiative developed in a rural healthcare setting.	Healthcare professionals, including nurses, physicians and paramedics involved in emergency care. The exact number of participants and their demographic characteristics were not reported in the source.	Eight simulation sessions on CPA management were delivered using the Anesoft Advanced Cardiovascular Life Support (ACLS) simulation software. During each session, team leaders were designated and team roles and responsibilities were reviewed before the scenario began.	Interdisciplinary simulation was found to enhance healthcare professionals’ confidence, strengthen team cohesion and provide valuable opportunities for critical reflection, constructive feedback and experiential learning based on previous clinical experiences.
O’Neill et al. (Canada, 2018) [50]	To develop a taxonomy of team SA and a reliable instrument for its assessment and construct validation.	Instrument-development and validation study conducted in three phases: development of an SA taxonomy, construction of an observational scoring instrument and assessment of its construct validity.	Phase 1 involved an interprofessional panel of 13 experts, including physicians, nurses and respiratory therapists. Phase 3 involved 242 practising healthcare professionals organised into 42 teams: 81 physicians, 105 nurses, 2 nurse practitioners and 54 respiratory therapists.	A literature review and expert consultation identified seven observable SA behaviours, which informed a scoring instrument classifying behaviours as positive, negative or absent. Validation was conducted in four university-affiliated paediatric hospitals and included theoretical and video-based training, four 15 min CPR simulations with repeated SA assessments, and post-simulation debriefing.	Team SA was characterised as a multidimensional construct comprising seven observable behaviours related to communication, role clarification and team decision-making. During validation, trained teams demonstrated better maintenance and recovery of SA than untrained teams, particularly when exposed to distractions.
Perez (USA, 2014) [51]	To develop and implement interdisciplinary team training in a simulated clinical setting to improve the response to CPA.	Descriptive study involving simulation-based training and a comparative analysis of performance before and after the intervention using validated scales.	Hospital emergency team comprising physicians, nurses and respiratory therapists; the number and demographic characteristics of participants were not reported.	Fortnightly one-hour in situ simulation sessions, comprising 10 min of simulation followed by 30–40 min of debriefing.	Interdisciplinary simulation was associated with significant improvements in resuscitation team performance, including teamwork and role clarification, supported by structured debriefing and performance assessment. The programme also identified skills deficits and organisational problems, contributing to improvements in CPA response processes and recognition of the nurse’s role in the initial leadership of the resuscitation team.
Popov et al. (USA, 2025) [52]	To analyse and compare communication skills and cognitive processes between expert and novice CPR team leaders.	Mixed-methods study using post-simulation interviews.	12 healthcare professionals: 7 Advanced Life Support instructors with leadership experience (experts) and 5 Advanced Life Support-trained participants with limited leadership experience (novices).	Standardised simulations using virtual reality were conducted, followed by individual interviews to analyse the NTS used throughout the different phases of CPR.	Experts demonstrated better NTS performance than novices, particularly in leadership, communication, teamwork, decision-making and task allocation. The authors suggested that virtual reality simulation may support the identification and development of these skills and the progressive acquisition of proficiency among CPR team leaders.
Renna et al. (Canada, 2016) [53]	To evaluate the impact of the Cognitive Aids for Role Definition (CARD) protocol on interprofessional crisis management during simulated intraoperative CPR.	Sequential mixed-methods study incorporating qualitative and quantitative analyses.	16 interprofessional operating room teams comprising anaesthetists, nurses, attending physicians and surgeons. The total number of individual participants and their demographic characteristics were not reported in the source.	Three simulation scenarios were completed: without the CARD protocol, with the protocol, and with the protocol plus additional training. Competency retention was assessed six months later.	The CARD protocol was reported to improve role clarity and team coordination, while promoting professional autonomy and contributing to a more structured and organised resuscitation environment.
Rovamo et al. (Finland, 2015) [54]	To evaluate whether prior instruction in NTS improves teamwork performance during simulated neonatal emergencies.	Experimental quantitative study employing a two-group design comprising intervention and control groups.	99 physicians and nurses allocated to an intervention group (62 participants; 9 teams) or control group (37 participants; 6 teams).	Both groups participated in high-fidelity in situ simulations of neonatal emergencies, including CPR, followed by structured debriefing. The intervention group additionally received a one-hour interactive NTS educational session before simulation.	No statistically significant differences in observed NTS were identified between the intervention and control groups, indicating that prior NTS instruction did not result in significant improvements. The findings suggest that a single, stand-alone NTS educational session may be insufficient to produce measurable improvements, reinforcing the importance of continuous, integrated simulation-based training. The authors also emphasised team leader profile and experience as key determinants of team performance, particularly during CPR.
Sawyer et al. (USA, 2013) [55]	To assess the impact of TeamSTEPPS interprofessional training on the development of NTS during neonatal resuscitation.	Prospective study assessing NTS before and after interprofessional training.	42 healthcare professionals: 10 physicians (4 specialists and 6 residents), 29 nurses and 3 respiratory therapists.	TeamSTEPPS training was delivered, complemented by simulation scenarios involving CPR, followed by structured debriefing sessions.	TeamSTEPPS training significantly improved all assessed dimensions of NTS, namely leadership, situational monitoring, mutual support and communication. The authors reported improvements in teamwork following interprofessional simulation and suggested that this approach may have implications for patient safety during CPR.
Undre et al. (UK, 2007) [56]	To develop, implement and evaluate a surgical crisis simulation module addressing technical and NTS, individual and team performance, and training gaps related to human factors and collaborative teamwork.	Cross-sectional observational study based on high-fidelity simulation.	80 healthcare professionals organised into 20 multidisciplinary teams, each comprising a surgeon, anaesthetist, nurse and operating department practitioner.	Simulation-based training using realistic intraoperative crisis scenarios, including CPR, conducted in a fully equipped operating theatre. Each 15 min scenario involved structured performance assessment and was followed by debriefing.	Teams demonstrating clear leadership and structured communication performed better, and participants reported greater SA following simulation. The authors concluded that simulation reinforced NTS, particularly communication, leadership and team coordination, with potential benefits for patient safety.
Wang et al. (Taiwan, 2019) [57]	To assess whether the implementation of low-fidelity in situ simulations contributed to reducing the incidence of unexpected CPA on a hospital ward over a four-year period.	Retrospective observational study conducted in Taiwan between 2012 and 2015.	84 in situ simulation sessions, each involving one intern, two junior residents and four nurses. Across the study period, 53 nurses participated, 16 of whom acted as simulation leaders at least once; other clinical team members participated as observers.	The in situ simulations were conducted fortnightly using low-fidelity manikins. Each 30 min session comprised a 5 min briefing, a 10 min simulation and a 15 min debriefing focused primarily on crisis resource management skills, including communication, teamwork, SA and decision-making.	The authors reported that in situ simulation reduced the incidence of CPA. Although no consistent improvement was observed in teams’ crisis resource management skills, simulation was reported as potentially useful in promoting a culture of safety, teamwork, communication and the early detection of clinical deterioration.
Wu et al. (USA, 2017) [58]	To develop and evaluate an interactive visual system providing real-time support to medical emergency teams during critical situations, including CPR, to facilitate adherence to clinical guidelines, improve SA and clinical task tracking, and support team coordination and collaboration in high-cognitive-demand environments.	Technological development study focused on participatory design and testing in a simulated clinical environment, including CPR scenarios.	Interprofessional team from a paediatric hospital. The exact number of participants and their demographic characteristics were not reported in the source.	Development of an interactive visual system informed by observations and interviews in real emergency settings, designed to display clinical guidelines, task status and real-time patient data and support time and priority management. The system was subsequently tested in a simulated clinical environment.	The system was reported as potentially reducing cognitive workload and supporting team performance. Participants perceived improvements in SA, communication and team coordination, with reduced need to recall cognitively complex steps during stressful situations. The authors suggested that the system may indirectly support communication, leadership and SA, while recommending further research to assess its direct impact on NTS.
Yun et al. (Republic of Korea, 2024) [59]	To examine the effect of structured team communication training on communication clarity and clinical competence during CPR by integrating a team communication programme based on standardised communication tools into an existing Advanced Life Support course.	Randomised controlled trial.	115 healthcare professionals: 73 physicians and 42 nurses.	Participants completed the Korean Advanced Life Support course, which included simulated CPA scenarios. The control group received the conventional course, while the intervention group additionally received TeamSTEPPS-based interprofessional team communication training, incorporating standardised communication tools during the simulations.	No significant differences in communication clarity were observed between the intervention and control groups. The greatest improvement was observed in SA, while decision-making also improved significantly among participants in the intervention group. The findings suggest that interprofessional team communication training integrated into simulated CPR scenarios may represent an effective strategy for enhancing the clinical competence of emergency care teams, particularly through the development of NTS.

## Data Availability

No new primary data were generated in this study. The data supporting the findings of this scoping review were obtained from the published sources cited in the manuscript and are presented within the article and its Supplementary Materials.

## Supplementary Materials

The following supporting information can be downloaded at https://www.mdpi.com/article/10.3390/nursrep16100364/s1, Supplementary File S1: Completed PRISMA-ScR checklist; Supplementary File S2: Complete database search strategies; Supplementary File S3: Data-charting form; Supplementary File S4: List of full-text articles excluded, with reasons for exclusion; Table S1: Data extraction tool developed for this Scoping Review.

## Public Involvement Statement

No public involvement in any aspect of this research.

## Guidelines and Standards Statement

This scoping review was conducted in accordance with the JBI methodological guidance for scoping reviews [18] and reported in accordance with the Preferred Reporting Items for Systematic Reviews and Meta-Analyses Extension for Scoping Reviews (PRISMA-ScR) reporting guideline [85,86].

## Abbreviations

The following abbreviations are used in this manuscript:

ACLS	Advanced Cardiovascular Life Support
CARD	Cognitive Aids for Role Definition
CPA	Cardiopulmonary Arrest
CPR	Cardiopulmonary Resuscitation
IHCA	In-Hospital Cardiac Arrest
MeSH	Medical Subject Headings
NTS	Non-Technical Skills
ODP	Operating Department Practitioner
PRISMA-ScR	Preferred Reporting Items for Systematic Reviews and Meta-Analyses Extension for Scoping Reviews
SA	Situational Awareness
TeamSTEPPS	Team Strategies and Tools to Enhance Performance and Patient Safety
TS	Technical Skills
UK	United Kingdom
USA	United States of America

## Appendix A. MeSH Terms and Alternative Terms

**Table A1:** MeSH descriptors, entry terms and natural-language terms used in the development of the search strategy. (*) was used as the truncation operator.

MeSH Terms/Natural Language Words	MeSH Terms/Alternative Terms
Simulation	Interactive Learning Debriefing
Soft Skill *	Non-Technical Skill * Clinical Competenc * Clinical Skill * Communication Decision Making Leadership Influentials Time Management Awareness *
Patient Care Team	Multidisciplinary Care Team Healthcare Team Medical Care Team Interdisciplinary Health Team Cooperative Behavior Compliant Behavior
Resuscitation *	Cardiopulmonary Resuscitation Heart Arrest Cardiac Arrest Cardiac Massage Heart Massage Cardiopulmonary Arrest Cardiac Life Support Asystole
