# Supplementary material for: Simulation in the Development of Non-Technical Skills in Cardiopulmonary Resuscitation: A Scoping Review
Source: Nurs Rep. 2026 Oct 13;16(10):364. doi: 10.3390/nursrep16100364 (PMC13708207; doi:10.3390/nursrep16100364)
Supplement: Supplementary file 1 [file nursrep-16-00364-s001.zip › File S2 Complete database search strategies.pdf]

Complete Database Search Strategies (EBSCOhost Interface)

1. MEDLINE (via EBSCOhost)

- **Database Name:** MEDLINE Complete (Medical Literature Analysis and Retrieval System Online)
- **Interface/Provider:** EBSCOhost
- **Date of Search:** July 2026
- **Search Mode**: Proximity
- **Fields Searched**: All Fields
- **Controlled-vocabulary headings explicitly incorporated into the search syntax:** None
- **Field Codes Explicitly Entered**: None
- **Boolean Operators**: AND; OR
- **Truncation**: * (asterisk)
- **Limits/Filters Applied:** Full-text availability
- **Date Restrictions:** None
- **Total Results:** 80
- **Search Strategy:**
    - S1 (Simulation OR Interactive Learning OR Debriefing)
    - S2 (Soft Skill* OR Non-Technical Skill* OR Clinical Competenc* OR Clinical Skill* OR Communication OR Decision Making OR Leadership OR Influentials OR Time Management OR Awareness*)
    - S3 (Patient Care Team OR Multidisciplinary Care Team OR Healthcare Team OR Medical Care Team OR Interdisciplinary Health Team OR Cooperative Behavior OR Compliant Behavior)
    - S4 (Resuscitation* OR Cardiopulmonary Resuscitation OR Heart Arrest OR Cardiac Arrest OR Cardiac Massage OR Heart Massage OR Cardiopulmonary Arrest OR Cardiac Life Support OR Asystole)
    - **Final Combination:** S1 AND S2 AND S3 AND S4

2. CINAHL (via EBSCOhost)

- **Database Name:** CINAHL (Cumulative Index to Nursing and Allied Health Literature)
- **Interface/Provider:** EBSCOhost
- **Date of Search:** July 2026
- **Search Mode**: Proximity
- **Fields Searched**: All Fields
- **Controlled-vocabulary headings explicitly incorporated into the search syntax:** None
- **Field Codes Explicitly Entered**: None

- **Boolean Operators**: AND; OR
- **Truncation**: * (asterisk)
- **Limits/Filters Applied:** Full-text availability
- **Date Restrictions:** None
- **Total Results:** 35
- **Search Strategy:**
  - S1 (Simulation OR Interactive Learning OR Debriefing)
  - S2 (Soft Skill* OR Non-Technical Skill* OR Clinical Competenc* OR Clinical Skill* OR Communication OR Decision Making OR Leadership OR Influentials OR Time Management OR Awareness*)
  - S3 (Patient Care Team OR Multidisciplinary Care Team OR Healthcare Team OR Medical Care Team OR Interdisciplinary Health Team OR Cooperative Behavior OR Compliant Behavior)
  - S4 (Resuscitation* OR Cardiopulmonary Resuscitation OR Heart Arrest OR Cardiac Arrest OR Cardiac Massage OR Heart Massage OR Cardiopulmonary Arrest OR Cardiac Life Support OR Asystole)
  - **Final Combination:** S1 AND S2 AND S3 AND S4

3. Nursing & Allied Health Collection (via EBSCOhost)

- **Database Name:** Nursing & Allied Health Collection
- **Interface/Provider:** EBSCOhost
- **Date of Search:** July 2026
- **Search Mode**: Proximity
- **Fields Searched**: All Fields
- **Controlled-vocabulary headings explicitly incorporated into the search syntax:** None
- **Field Codes Explicitly Entered**: None
- **Boolean Operators**: AND; OR
- **Truncation**: * (asterisk)
- **Limits/Filters Applied:** Full-text availability
- **Date Restrictions:** None
- **Total Results:** 7
- **Search Strategy:**
  - S1 (Simulation OR Interactive Learning OR Debriefing)
  - S2 (Soft Skill* OR Non-Technical Skill* OR Clinical Competenc* OR Clinical Skill* OR Communication OR Decision Making OR Leadership OR Influentials OR Time Management OR Awareness*)
  - S3 (Patient Care Team OR Multidisciplinary Care Team OR Healthcare Team OR Medical Care Team OR Interdisciplinary Health Team OR Cooperative Behavior OR Compliant Behavior)

    - S4 (Resuscitation* OR Cardiopulmonary Resuscitation OR Heart Arrest OR Cardiac Arrest OR Cardiac Massage OR Heart Massage OR Cardiopulmonary Arrest OR Cardiac Life Support OR Asystole)
    - **Final Combination:** S1 AND S2 AND S3 AND S4

4. Cochrane Central Register of Controlled Trials (CENTRAL) (via EBSCOhost)

- **Database Name:** Cochrane Central Register of Controlled Trials
- **Interface/Provider:** EBSCOhost
- **Date of Search:** July 2026
- **Search Mode**: Proximity
- **Fields Searched**: All Fields
- **Controlled-vocabulary headings explicitly incorporated into the search syntax:** None
- **Field Codes Explicitly Entered**: None
- **Boolean Operators**: AND; OR
- **Truncation**: * (asterisk)
- **Limits/Filters Applied:** Full-text availability
- **Date Restrictions:** None
- **Total Results:** 16
- **Search Strategy:**
    - S1 (Simulation OR Interactive Learning OR Debriefing)
    - S2 (Soft Skill* OR Non-Technical Skill* OR Clinical Competenc* OR Clinical Skill* OR Communication OR Decision Making OR Leadership OR Influentials OR Time Management OR Awareness*)
    - S3 (Patient Care Team OR Multidisciplinary Care Team OR Healthcare Team OR Medical Care Team OR Interdisciplinary Health Team OR Cooperative Behavior OR Compliant Behavior)
    - S4 (Resuscitation* OR Cardiopulmonary Resuscitation OR Heart Arrest OR Cardiac Arrest OR Cardiac Massage OR Heart Massage OR Cardiopulmonary Arrest OR Cardiac Life Support OR Asystole)
    - **Final Combination:** S1 AND S2 AND S3 AND S4
