# Supplementary material for: Simulation in the Development of Non-Technical Skills in Cardiopulmonary Resuscitation: A Scoping Review
Source: Nurs Rep. 2026 Oct 13;16(10):364. doi: 10.3390/nursrep16100364 (PMC13708207; doi:10.3390/nursrep16100364)
Supplement: Supplementary file 1 [file nursrep-16-00364-s001.zip › File S3 Data-charting form.pdf]

## Data extraction / Data-charting Form

The research team developed a data extraction tool based on the template recommended by the Joanna Briggs Institute (JBI) for data extraction in scoping reviews. Accordingly, the tool included information relating to the author(s), year of publication, country of origin (i.e., where the source was published), study objective, methodology, participants/population, as well as the type of intervention implemented and related details, together with the study results and associated information [32].

Prior to commencing the data extraction process, a pilot test of the extraction tool was conducted by the reviewers using a sample of 3 of the 29 included sources of evidence. This process enabled the reviewers to assess the clarity, relevance, and adequacy of the extracted variables and to make modifications to the tool, where necessary.

Following the pilot test, adjustments were made to the data extraction tool. Specifically, it was decided to incorporate information regarding the type of intervention implemented and related details, as well as the study results and associated information, within the section entitled key findings that relate to the scoping review question.

The final version of the data extraction tool, following revision and pilot testing, is presented below in Table S1.

**Table S1.** Data extraction tool developed for this Scoping Review.

| Author(s) (Country/Year) [Reference] | Objective | Methodology | Participants | Key Findings | |
|---|---|---|---|---|---|
| | | | | Intervention | Conclusions |
| - | - | - | - | - | - |
