# Supplementary material for: Simulation in the Development of Non-Technical Skills in Cardiopulmonary Resuscitation: A Scoping Review
Source: Nurs Rep. 2026 Oct 13;16(10):364. doi: 10.3390/nursrep16100364 (PMC13708207; doi:10.3390/nursrep16100364)
Supplement: Supplementary file 1 [file nursrep-16-00364-s001.zip › File S4 List of full-text articles excluded, with reasons for exclusion.pdf]

List of Full-Text Articles Excluded with Reasons

Reason: Full-Text Not Available In English, Portuguese Or Spanish

Article Name	Database	Language
Implementierung Eines Regelmäßigen Reanimationstrainings An Einem Druckkammerzentrum	CINAHL	German
Post-Resuscitation Talk In The Intensive Care Unit: Living Interprofessionalism-A Must Have!	MEDLINE	German
Nurses' Cardiopulmonary Resuscitation Performance During The First 5 Minutes In In-Situ Simulated Cardiac Arrest	MEDLINE	Korean
Implementation Of Regularly Performed Resuscitation Training At A Hyperbaric Treatment Center	MEDLINE	German
Using Team Resource Management And Situational Simulation To Improve The Resuscitation Performance Completion Rate	MEDLINE	Chinese
Crew Resource Management And Simulator Training In Acute Stroke Therapy	MEDLINE	German

Reason: Did Not Address Cardiopulmonary Resuscitation Situations

Article Name	Database
A Collaborative Partnership For Improving Newborn Safety: Using Simulation For Neonatal Resuscitation Training	CINAHL
A Multidisciplinary Simulated Teaching Programme On Treatment Escalation Planning In The Context Of Frailty	CINAHL
Training Leaders In Trauma Resuscitation: Teacher And Learner Perspectives On Ideal Methods	CINAHL
Using "The Burns Suite" As A Novel High Fidelity Simulation Tool For Interprofessional And Teamwork Training	CINAHL
Integrating The Electronic Health Record Into High-Fidelity Interprofessional Intensive Care Unit Simulations	CINAHL
Evaluation Of Two Strategies For Debriefing Simulation In The Development Of Skills For Neonatal Resuscitation: A Randomized Clinical Trial.	COCHRANE
Peer-Assisted Learning After Onsite, Low-Dose, High-Frequency Training And Practice On Simulators To Prevent And Treat Postpartum Hemorrhage And Neonatal Asphyxia: A Pragmatic Trial In 12 Districts In Uganda	COCHRANE
Impact Of Video Recordings Review With Structured Debriefings On Trauma Team Performance: A Prospective Observational Cohort Study	MEDLINE

Translatability And Validation Of Non-Technical Skills Scale For Trauma (T-NOTECHS) For Assessing Simulated Multi-Professional Trauma Team Resuscitations	MEDLINE
Efficacy Of Simulation-Based Trauma Team Training Of Non-Technical Skills. A Systematic Review	MEDLINE
Team Talk And Team Activity In Simulated Medical Emergencies: A Discourse Analytical Approach	MEDLINE
Preparing Austere Maritime Surgical Teams For Deployment During The COVID-19 Global Pandemic: Is It Time To Change The Training Pipeline?	MEDLINE
Trauma Simulation In Bilingual Canada: Insurmountable Barrier Or Unexpected Strength? Insights From The First Bilingual S.T.A.R.T.T. Course	MEDLINE
Enhancing Nurse-Physician Collaboration Using Pediatric Simulation	MEDLINE
Surgical Team Assessment Training: Improving Surgical Teams During Deployment	MEDLINE
Trauma Resuscitation Evaluation Times And Correlating Human Patient Simulation Training Differences-What Is The Standard?	NURSING & ALLIED

Reason: Did Not Address Non-Technical Skills

Article Name	Database
Identifying And Managing Latent Safety Threats Through A Zone-Wide Emergency Department In-Situ Multidiscipline Simulation Program: A Quality Improvement Project	CINAHL
Development Of A "First Five Minutes" Program To Improve Staff Response To Pediatric Codes	MEDLINE

Reason: Did Not Address A Simulation Context

Article Name	Database
Maternal Collapse In Pregnancy	CINAHL
The Impact Of A Fellow-Driven Debriefing Program After Pediatric Cardiac Arrests	MEDLINE
Situational Awareness Huddles in a Pediatric Cardiac Intensive Care Unit During the COVID-19 Pandemic	NURSING & ALLIED

Reason: Included Undergraduate Nursing And/Or Medical Students

Article Name	Database
Flipped Classroom: Improved Team Performance During Resuscitation Training Through Interactive Pre-Course Content - A Cluster-Randomised Controlled Study	COCHRANE
Effect Of CRM Team Leader Training On Team Performance And Leadership Behavior In Simulated Cardiac Arrest Scenarios: A Prospective, Randomized, Controlled Study	MEDLINE
Rating The Quality Of Teamwork-A Comparison Of Novice And Expert Ratings Using The Team Emergency Assessment Measure (TEAM) In Simulated Emergencies	MEDLINE
NRTS: A Client-Server Architecture For Supporting Education In A Neonatal Resuscitation Simulation Scenario	MEDLINE
Leadership And Teamwork In Trauma And Resuscitation	MEDLINE
Interdepartmental Collaboration For Simulation-Based Education: Obstetric Emergencies For Emergency Medicine	MEDLINE

Reason: Did Not Report an Eligible Participant Sample

Article Name	Database
Sensing and visualization tools for objective assessment and debriefing of high-risk neonatal resuscitation training scenarios.	MEDLINE

Reason: Constituted a Secondary Source That Did Not Report Original Data

Article Name	Database
Simulation in neonatal care: towards a change in traditional training?	MEDLINE
